# Molecular Classification Based on the Gene Expression Profiles in Canine Histiocytic Sarcoma Cells

**DOI:** 10.1111/vco.13071

**Published:** 2025-06-11

**Authors:** Hiroki Sakuma, Hirotaka Tomiyasu, Akiyoshi Tani, Yuko Goto‐Koshino, Makoto Bonkobara, Masaru Okuda

**Affiliations:** ^1^ Laboratory of Veterinary Internal Medicine, Department of Veterinary Medical Sciences, Graduate School of Agricultural and Life Sciences The University of Tokyo Bunkyo‐ku Tokyo Japan; ^2^ Veterinary Medical Center, Graduate School of Agricultural and Life Sciences The University of Tokyo Bunkyo‐ku Tokyo Japan; ^3^ Department of Veterinary Clinical Pathology Nippon Veterinary and Life Science University Musashino‐shi Tokyo Japan

**Keywords:** dog, intracellular signalling, molecular subtype, NF‐kB pathway, RNA‐sequencing

## Abstract

The molecular abnormalities of canine histiocytic sarcoma (CHS) remain to be elucidated. We previously revealed that the sensitivities to dasatinib and trametinib were significantly various among CHS cell lines, indicating the differences in underlying molecular abnormalities. In the present study, we performed RNA sequencing analysis using 11 CHS cell lines to investigate molecular classifications based on the gene expression profiles (GEPs). The clustering analysis showed that CHS cell lines were divided into two distinct clusters. The comparisons of GEPs between the clusters extracted 675 differentially expressed genes (DEGs), and these DEGs were enriched with those related to the regulations of inflammatory responses. Among these DEGs, differences in the expressions of *CCL3*, *CCL4*, *CCL7*, *CLEC7A*, and *TLR4* genes between the two groups were confirmed by RT‐qPCR. Since no significant difference in the activation status of Akt and ERK pathways was observed between the two groups, the NF‐κB pathway was focused on and its activation status was examined in the cell lines. As a result, cell lines belonging to one cluster showed nuclear translocation of the p65 protein together with increased release of CCL5 protein, which is a target molecule of the NF‐κB pathway, in a cell culture supernatant. These results suggested that the molecular pathology of CHS cells might be divided into two categories depending on the activation status of the NF‐κB pathway, and it is necessary to establish precision medicine for each molecular subtype of CHS.

## 1 Introduction

Canine histiocytic sarcoma (CHS) is a rare and aggressive tumour that originates from interstitial dendritic cells and macrophages [1]. CHS occurs in various organs including bone marrow, liver, lung, lymph node, skin, and spleen [1], and it is treated with surgery, radiation therapy, and chemotherapy [2]. Various anticancer drugs including lomustine have been applied for CHS cases, showing antitumor effects in only a limited proportion of cases [3, 4, 5, 6, 7, 8]. Therefore, novel effective treatments for CHS need to be established.

In human medicine, the presence of the BRAF V600E mutation and activation of ERK have been reported in histiocytic sarcoma, and the antitumor effect of the combination of trametinib and dabrafenib has been investigated [9, 10, 11]. In a previous study, we also showed that the ERK and Akt pathways were activated in CHS [12]. This finding was concordant with the previous observations that indicated the effectiveness of dasatinib, a proto‐oncogene non‐receptor tyrosine kinase (Src) inhibitor, and trametinib, a mitogen‐activated protein kinase (MEK) inhibitor against CHS cells [13, 14, 15, 16]. However, we recently found that the blockade of these pathways exhibited growth inhibition in some cell lines but not in others despite the ERK and Akt pathways being similarly activated [17]. These results indicated underlying divergent molecular pathologies of CHS cells, which should be associated with the different sensitivities to the molecular‐targeting drugs.

Multiple studies have reported genetic aberrations in CHS cases. *PTPN11* gene mutations have been frequently detected in CHS that occurred in Bernese Mountain Dog and Golden Retriever [18, 19, 20], while they are rarely found in Flat‐Coated Retriever [19]. Additionally, chromosomal abnormalities such as deletions or copy number increases have been reported in the region near the *cyclin‐dependent kinase inhibitor 2A* or *2B* (*CDKN2A/2B*), *retinoblastoma 1* (*RB1*), and *phosphatase and tensin homologue* (*PTEN*) genes [21, 22], but these abnormalities were observed in only a minority of the CHS cases. Moreover, it was recently reported that the expression of *Secreted Phosphoprotein 1* (*SPP1*) gene was upregulated in CHS cases, but its expression levels at the protein level varied among cases possibly depending on the locations of the lesions [23]. These findings in CHS cases also indicated the molecular pathological heterogeneity in CHS, which could be related to the diverse response to molecular‐targeting drugs.

Together, these findings indicate that CHS cells could be subclassified based on gene expression profiles (GEPs). We performed a comprehensive analysis of GEPs using CHS cell lines by RNA‐sequencing analysis in the present study, and we found that CHS cells could be classified based on the activation status of the NF‐κB pathway.

## 2 Materials and Methods

### 2.1 Cell Lines and Cell Culture

Eleven CHS cell lines were used in this study. CHS2, CHS3, CHS4, CHS5, CHS6, CHS7, CHS8 (ROMA), and MHT2 were previously established from dogs with CHS at Nippon Veterinary and Life Science University (Table 1) [24]. DHS1 and DHS2 were established from CHS patient dogs at the University of Tokyo and used in our previous study (Table 1) [12]. DH82 was established from a dog with hemophagocytic CHS (Table 1) [25].

**TABLE 1:** Canine histiocytic sarcoma cell lines used in this study.

Cell lines	Breed	Age	Sex	Diagnosis	Origin tissues	Mutations in *PTPN11* gene
CHS2	Shetland sheepdog	8Y	Male	Disseminated HS	Skin	
CHS3	Bernese mountain dog	6Y	Male	Disseminated HS	Skin	E76Q
CHS4	Flat coated retriever	6Y	Female	Solitary HS	Synovial	
CHS5	Golden retriever	8Y	Male	Solitary HS	Synovial	
CHS6	Bernese mountain dog	7Y	Male	Solitary HS	Lymph node	E76A
CHS7	Flat coated retriever	8Y	Male	Solitary HS	Synovial	
CHS8 (ROMA)	N/A	N/A	N/A	N/A	Skin	G503V
DH82	Golden retriever	10Y	Male	Disseminated HS (hemophagocytic)	Spleen	G503V
DHS1	Shiba‐dog	12Y	Female	Disseminated HS	Lower jaw	
DHS2	Miniature schnauzer	6Y	Female	Disseminated HS	Pleural effusion	G503V
MHT2	N/A	N/A	N/A	N/A	N/A	

Abbreviation: N/A: Not available.

All CHS cell lines were cultured at 37°C in Dulbecco's modified Eagle's medium (DMEM, Wako) containing 10% fetal bovine serum (Biowest) and 1% penicillin–streptomycin (Wako) in a humidified atmosphere containing 5% CO_2_.

### 2.2 Cell Line Authentication Statement

CHS2 to CHS7 were authenticated as CHS cell lines as previously reported [24]. The remaining cell lines were confirmed as being derived from CHS through immunohistochemical staining of the original lesions. All CHS cell lines used in this study were confirmed to be free of mycoplasma contamination, as verified by a PCR‐based Mycoplasma Detection Kit (TaKaRa).

### 2.3 Extraction of Total RNA

Total RNA was extracted from the cell lines using the Allprep RNA/DNA Mini Kit (Qiagen) following the manufacturer's protocol. RNA samples were immediately frozen and stored at‐80°C until use.

### 2.4 RNA Sequencing Analysis

RNA integrity was examined using an Agilent 2100 Bioanalyzer (Agilent Technologies), and the RNA integrity number was confirmed to be > 9.0 (range; 9.1–9.7, Table S1). Messenger RNA (mRNA) was isolated from total RNA using the NEBNext poly(A) mRNA Magnetic Isolation Module (New England Biolabs) and reverse transcribed to generate complementary DNA (cDNA). A library generated by the NEBNext Ultra 2 Directional RNA Library Prep kit (New England Biolabs) was subjected to paired‐end sequencing using the Illumina NovaSeq 6000 (Illumina). The quality of each sample read was assessed using the fastQC software (version 0.11.9). The Fastp software (version 0.20.1) was used to perform adaptor trimming for each FASTQ file. The reads with a sequence length of < 20 base pairs or 20 < sequences designated as ‘N' were excluded from the analysis. Furthermore, a single base pair was removed from the tail of the sequence. The trimmed reads were mapped to the reference genome (CanFam3.1 annotated by Ensembl 104) using the STAR software (Version 2.7.3a) [26] and the mapped reads were quantified gene‐by‐gene using the RSEM software (version 1.3.1) [27]. The BAM files generated by RSEM were used to calculate the mapping rate using samtools software (version 1.3) and also used as the input for the subsequent analysis [28]. We also evaluated the mutations in *PTPN11* gene in each cell line using the obtained BAM files by Integrative Genomics Viewer (IGV) application [29]. The obtained gene count data was normalised by iDEGES/edgeR and calculated for average‐linkage clustering after Spearman's rank correlation coefficient using the TCC (ver. 1.32.0) [30], a tool integrated into the R (ver. 4.1.3) package. The differentially expressed genes (DEGs) were extracted based on a false discovery rate (FDR) of < 0.2 in the comparisons between the clusters. To explore the biological mechanisms and pathways associated with the extracted DEGs, gene ontology (GO) enrichment analysis [31, 32] and Kyoto Encyclopedia of Genes and Genomes (KEGG) pathway analysis [33] were performed using the Database for Annotation, Visualisation and Integrated Discovery (DAVID, ver. 2024q4) [34], and the terms were extracted based on an FDR of < 0.05.

### 2.5 Reverse Transcription‐Quantitative Polymerase Chain Reaction (RT‐qPCR)

The total RNAs extracted from 11 CHS cell lines were reverse transcribed using ReverTra Ace RT Master Mix (Toyobo) according to the manufacturer's instructions. The relative amounts of each gene mRNA were quantified using TB Green Premix ExTaq II (Tli RNaseH plus) (Takara) on a StepOnePlus Real‐Time PCR system (Applied Biosystems). The sequences of primer pairs are shown in Table S2. The gene expression levels of each gene were calculated using the relative standard curve method, with *RPL32* serving as the internal control gene, as previously described [12]. All samples were measured in duplicate.

### 2.6 Immunocytochemistry

To generate a monolayer slide specimen, a cell suspension of 5 × 10^4^ cells was centrifuged at 500 rpm for 5 min in the Cytospin 4 (Thermo Fisher Scientific). After air drying, cells were fixed by immersion in 4% paraformaldehyde (Fujifilm) for 15 min at room temperature. Cell permeabilization was performed by placing the cells in 0.25% (w/v) Triton‐X in phosphate‐buffered saline (PBS) for 10 min at room temperature. Blocking was performed by incubating with a 5% bovine serum albumin solution for 1 h at room temperature. Then, the cells were incubated with the primary antibody (rabbit anti‐NF‐κB p65 XP, D14E12; Cell Signalling Technology, diluted 1:500) at 4°C overnight. After washing, the cells were incubated with a secondary antibody (donkey anti‐rabbit IgG (H + L) highly cross‐adsorbed secondary antibody labelled by Alexa Fluor 488; Invitrogen, diluted 1:200) at room temperature for 2 h in the dark. After washing, the cells were incubated with Phalloidin conjugated with rhodamine X (FujiFilm) at room temperature for 30 min in the dark. For mounting and nuclear staining, Fluoromount‐G Mounting Medium, with 4′,6‐diamidino‐2‐phenylindole (DAPI) (Thermo Fisher Scientific) was added to the samples, and glass coverslips were placed on the slides. Images were captured using LSM 700 (Zeiss) at 20x magnification. Three images were taken for each slide, and all cell lines were examined in triplicate. The proportions of cells with p65 nuclear translocation were calculated by dividing the number of cells with p65 protein within the nucleus by total cell counts detected with DAPI.

### 2.7 Enzyme‐Linked Immuno Sorbent Assay (ELISA)

Each cell line was seeded in a 24‐well plate at 8 × 10^4^ cells/mL. After 72 h, the cell culture medium was collected, centrifuged at 1500 rpm for 10 min at 4°C, and the supernatant was stored at −80°C. After thawing, the amount of CCL5 protein was measured using the canine CCL5/RANTES DuoSet ELISA kit (R&D systems) according to the manufacturer's protocol.

### 2.8 Cell Viability Assay

Two thousand cells/well were seeded in a 96‐well plate and incubated in DMEM at 37°C for 24 h. Then, the cells were incubated for 72 h after replacing the medium with DMEM supplemented with QNZ, an NF‐κB specific inhibitor [35], dissolved in dimethyl sulfoxide (DMSO) at the concentration of 1 μM. The final concentration of DMSO in the growth medium was kept not to exceed 0.1% (v/v), and equal amounts of DMSO were added to the control conditions as well. After the incubation period, 10 μL of the cell counting kit‐8 (Dojindo) was added to each well, and cells were incubated for 2 h 30 min at 37°C. The absorbance in each well was measured at 450 nm single laser using Model 680 Microplate Reader (Bio‐Rad Laboratories), and viable cell counts were calculated. Cell viability was calculated as a ratio of viable cell count treated by QNZ to those of vehicle‐administered controls. These experiments were performed at least in triplicate.

### 2.9 Statistical Analysis

The association of the proportions of cells showing nuclear translocation of p65 with the cell viability after treatment with QNZ was examined using R software (https://www.R‐project.org/, ver.4.3.3).

## 3 Results

### 3.1 Clustering Analysis of 11 CHS Cell Lines Based on GEPs

**FIGURE 1:**
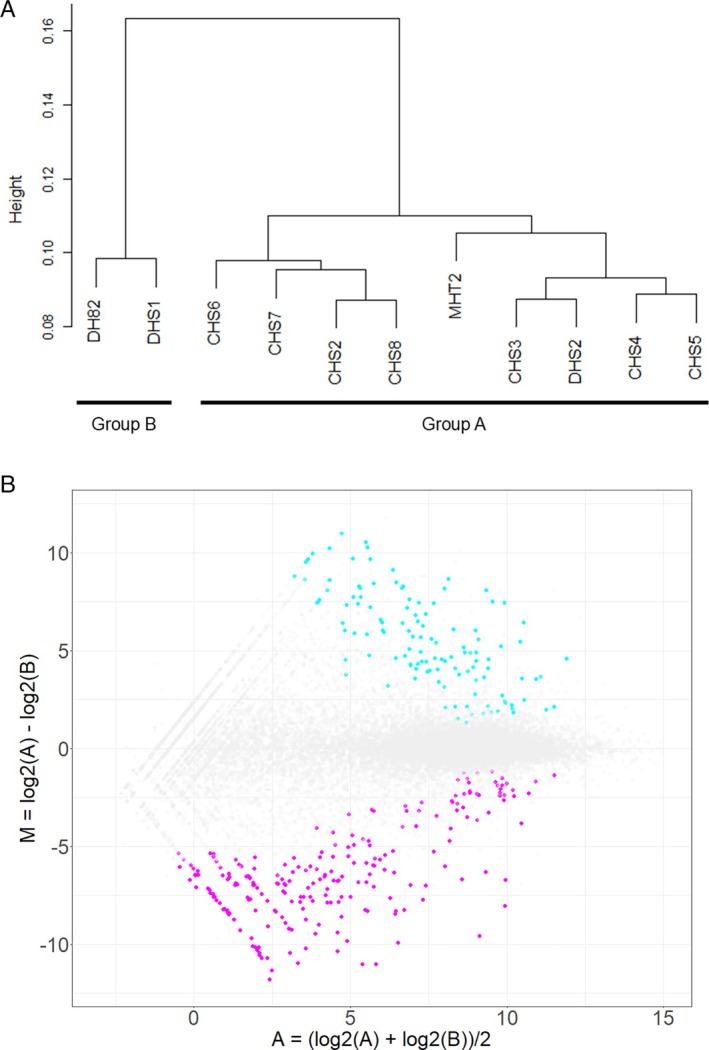
(A) Hierarchical clustering analysis with canine histiocytic sarcoma cell lines based on gene expression profiles. (B) M‐A plot with differentially expressed genes (DEGs) extracted by the comparisons between Group A and B. The points coloured in cyan represent the DEGs (283 genes) whose expressions were increased in Group A compared to Group B. The points coloured in magenta represent the DEGs (392 genes) whose expressions were decreased in Group A compared to Group B.

RNA‐sequencing analysis was performed using RNA extracted from 11 CHS cell lines. The median read count was 17.02 million reads (range; 13.68–21.29 million reads, Table S1), and the median mapping rate was 97.49% (range; 95.93%–97.82%, Table S1). In hierarchical clustering analysis, the CHS cell lines were distinctly divided into two groups (Figure 1A). Group A included CHS2, CHS3, CHS4, CHS5, CHS6, CHS7, CHS8, DHS2, and MHT2, and Group B included DHS1 and DH82. The comparisons of GEPs between Group A and B extracted 675 DEGs (Figure 1B). We could extract 283 DEGs upregulated in Group A (Table S3) and 392 DEGs downregulated in Group A (Table S4). Among the highly expressed genes in Group A compared to Group B, those with the smallest FDR values included *colony stimulating factor 1 receptor* (*CSF1R*) and *interleukin 10 receptor subunit alpha* (*IL10RA*); the former is associated with the macrophages production and function [36], and the latter is a receptor for IL‐10 and suppresses inflammation through the activation of the STAT pathway (Table 2) [37]. The lowly expressed genes in Group A with the smallest FDR values included *AXL*, which is a tyrosine kinase receptor associated with cell survival and proliferation [38], and fibroblast growth factor 19 (*FGF19*), which is included in the FGF family that regulates cell survival and proliferation (Table 3) [39].

**TABLE 2:** Differentially expressed genes upregulated in Group A compared to Group B. (Top 20).

ENSEMBL Gene ID	Gene symbol	A.value	M.value	FDR
ENSCAFG00000017712	*IGSF6*	4.326419105	8.603924243	0.00239598
ENSCAFG00000011210	*UNC93B1*	9.403405028	4.570061191	0.00392298
ENSCAFG00000016764	*SLC49A3*	5.310085814	8.192158157	0.003942194
ENSCAFG00000001522	*NCF4*	−8.33613348	17.06095941	0.003942194
ENSCAFG00000005415	*ARRB1*	5.066168932	9.704324393	0.004683303
ENSCAFG00000018219	*CSF1R*	9.915160752	7.446157352	0.005585338
ENSCAFG00000013314	*NCF2*	8.655537203	4.890104145	0.005892134
ENSCAFG00000008804	*MARCHF1*	5.332003964	7.728745883	0.007890471
ENSCAFG00000006704	*NFKBID*	8.750238931	3.941015269	0.008344793
ENSCAFG00000002283	*FGD3*	−8.33613348	14.8408213	0.008472585
ENSCAFG00000009680	*GCNT2*	3.965359004	7.502704535	0.008472585
ENSCAFG00000013819	*FMNL1*	9.010769064	4.469356155	0.008486983
ENSCAFG00000018540	*MYO1F*	5.633619585	9.669300697	0.008486983
ENSCAFG00000028607	*GMFG*	8.210071237	4.66525419	0.00911812
ENSCAFG00000015163	*CFP*	6.361356786	9.117553963	0.00987693
ENSCAFG00000003709	*CD37*	7.314747889	6.269219724	0.010263116
ENSCAFG00000010231	*KCNQ1*	5.490555932	10.55309839	0.010332457
ENSCAFG00000011943	*TRAF3IP3*	5.527250638	5.835662309	0.011240514
ENSCAFG00000003518	TLR4	6.8127693	7.197525128	0.013804638
ENSCAFG00000012844	*IL10RA*	6.440055568	4.628211937	0.013967289

*Note*: A.value: Average of Log_2_ value of average read count in each group, (Log2[Group A]+Log2[Group B])/2; Log_2_ value of average read count for each gene in the cell lines of Group A, Log_2_[Group B]: Log_2_ value of average read count for each gene in the cell lines of Group B. M.value: Difference in Log_2_ value of average read count between the cell lines of Group A and Group B, Log_2_[Group A]‐ Log_2_[Group B]: Log_2_ value of average read count for each gene in the cell lines of Group A, Log_2_[Group B]: Log_2_ value of average read count for each gene in the cell lines of Group B.

Abbreviation: FDR: False discovery rate.

**TABLE 3:** Representative differentially expressed genes downregulated in Group A compared to Group B. (Top 20).

ENSEMBL Gene ID	Gene symbol	A.value	M.value	FDR
ENSCAFG00000014752	*TFPI*	3.871129465	−14.0697219	3.93E‐07
ENSCAFG00000020432	*WLS*	5.370724707	−11.01639084	0.000256398
ENSCAFG00000009658	*TFAP2A*	2.495631145	−11.33430686	0.000364337
ENSCAFG00000015135	*LDB2*	2.409840486	−11.79192659	0.000364337
ENSCAFG00000005041	*AXL*	5.105307876	−12.64635737	0.000401385
ENSCAFG00000017343	*KCNK10*	3.312712549	−10.96846967	0.000401385
ENSCAFG00000016557	*EXD2*	9.050913251	−3.664523404	0.001186224
ENSCAFG00000006371	*NRG1*	6.717297145	−8.24134132	0.001186224
ENSCAFG00000000789	*WNT7B*	2.156960381	−10.69539264	0.001323311
ENSCAFG00000018381	*ESM1*	6.509970578	−9.934405069	0.002254759
ENSCAFG00000014322	*PCOLCE*	7.313582336	−7.71694293	0.002254759
ENSCAFG00000010693	*FGF19*	2.095551916	−10.57257571	0.002254759
ENSCAFG00000011907	*PCBP3*	1.837033202	−9.692402247	0.00283455
ENSCAFG00000001623	*SLC24A2*	2.09552978	−10.43900692	0.003065839
ENSCAFG00000012414	*FAM13C*	3.06883447	−10.45160625	0.00392298
ENSCAFG00000014812	COL3A1	4.576808117	−10.3466771	0.00392298
ENSCAFG00000010930	*COL16A1*	4.574994599	−9.391342823	0.00392298
ENSCAFG00000007590	*ZNF507*	3.57688643	−8.748351496	0.003942194
ENSCAFG00000011426	*PTGIS*	6.439251284	−8.301659495	0.004683303
ENSCAFG00000006634	*JAKMIP2*	1.473567951	−9.290180472	0.005447838

*Note*: A.value: Average of Log_2_ value of average read count in each group, (Log2[Group A]+Log2[Group B])/2; Log_2_ value of average read count for each gene in the cell lines of Group A, Log_2_[Group B]: Log_2_ value of average read count for each gene in the cell lines of Group B. M.value: Difference in Log_2_ value of average read count between cell lines of Group A and Group B, Log_2_[Group A]‐ Log_2_[Group B]: Log_2_ value of average read count for each gene in the cell lines of Group A, Log_2_[Group B]: Log_2_ value of average read count for each gene in the cell lines of Group B.

Abbreviation: FDR: False discovery rate.

### 3.2 Pathway Analysis Using DEGs Extracted by Comparison Between the Group A and B

The biological implications of DEGs extracted from the comparison between Group A and B were examined by GO and KEGG pathway analysis. In the category of biological process in the GO terms, 39 terms including “inflammatory response” and “extracellular matrix organization” were shown to be enriched with DEGs (Table S5). We also found that 21 terms including “plasma membrane” and “extracellular region” in the category of cellular component were enriched with DEGs (Table S6) and 8 terms including “extracellular matrix structural constituent” and “integrin binding” in the category of molecular function were enriched with DEGs (Table S7). In KEGG pathway analysis, we found that pathways including “phagosome” and “proteoglycans in cancer” were enriched with DEGs (Table S8). Among these terms and pathways, we decided to focus on those associated with inflammatory response based on its well‐established association with cancer progression.

### 3.3 Comparisons of Expression Levels of Genes Related to Inflammatory Response

**FIGURE 2:**
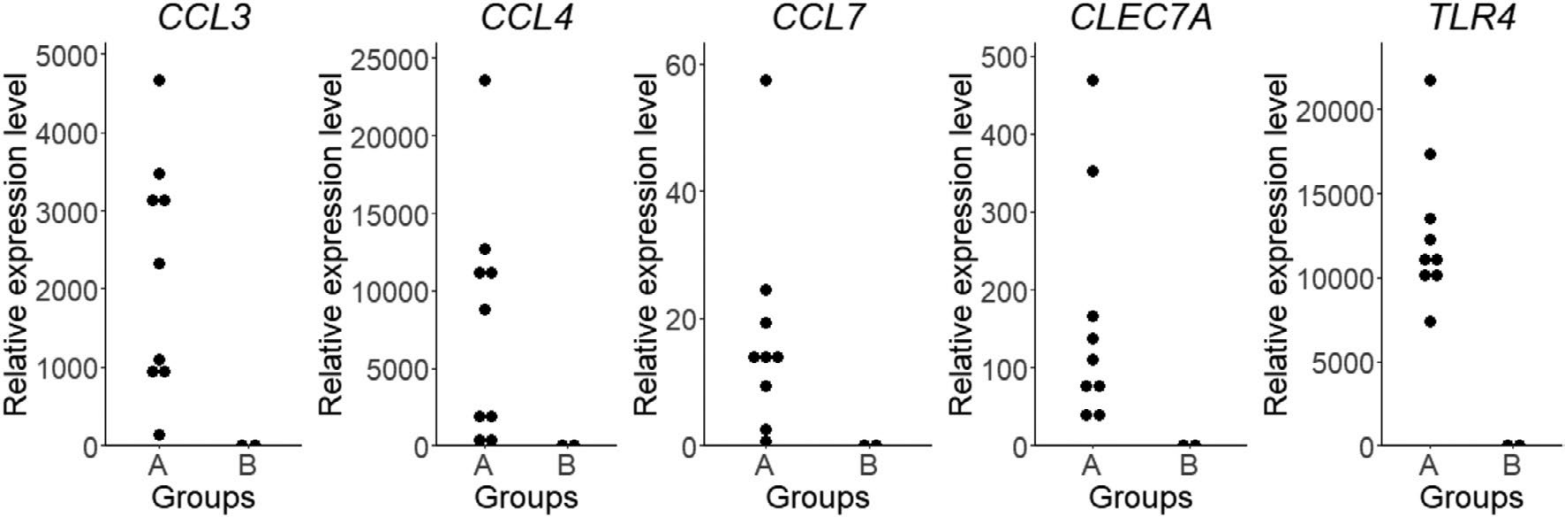
Comparisons of gene expression levels of *CCL3*, *CCL4*, *CCL7*, *CLEC7A*, and *TLR4* genes between Group A and B. Each experiment was conducted in triplicate.

Among the extracted DEGs, 42 genes were associated with the inflammatory response, and we decided to focus on the genes involved in Akt, ERK, and NF‐κB pathways, which have been previously reported as the activated pathways in CHS [12, 17, 40]. Consequently, C‐C motif chemokine ligand 3 (*CCL3*), C‐C motif chemokine ligand 4 (*CCL4*), C‐C motif chemokine ligand 7 (*CCL7*), C‐type lectin domain containing 7A (*CLEC7A*), and toll‐like receptor 4 (*TLR4*) were selected for further investigations. Consistent with the results in RNA‐sequencing analysis, RT‐qPCR analysis showed that the mRNA expression levels of *CCL3, CCL4, CCL7, CLEC7A*, and *TLR4* genes were increased in the cell lines of Group A (Figure 2).

### 3.4 Comparisons of the Activation Status of ERK, Akt, and NF‐κB Pathways Between Group A and B

**FIGURE 3:**
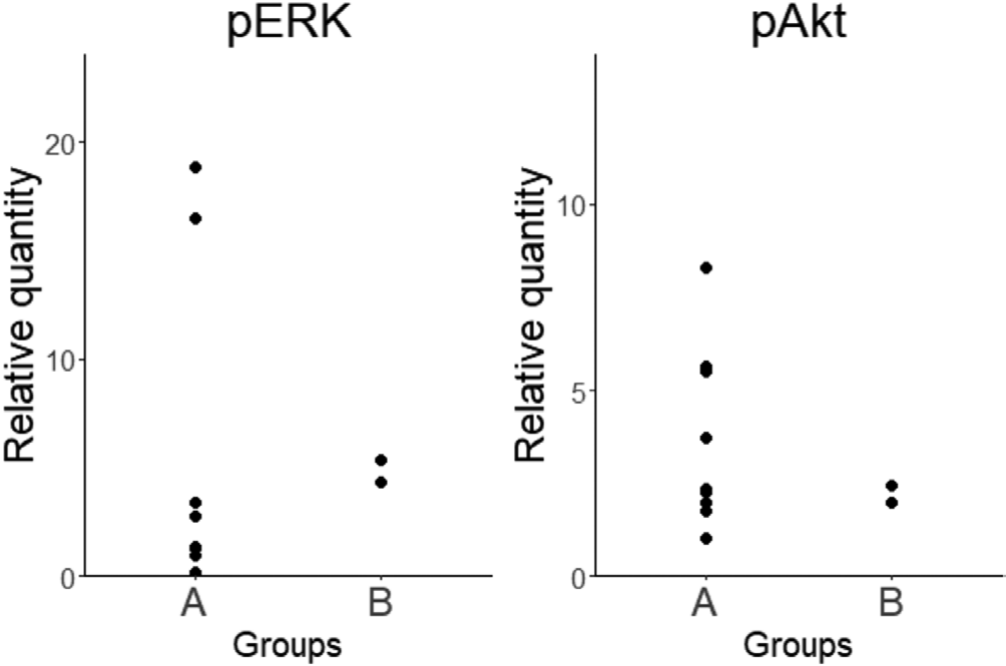
Comparisons of relative quantities of phosphorylated ERK protein and phosphorylated Akt proteins between Groups A or B with the quantity of DH82 using the data we have previously published [12]. Each experiment was conducted in triplicate.

**FIGURE 4:**
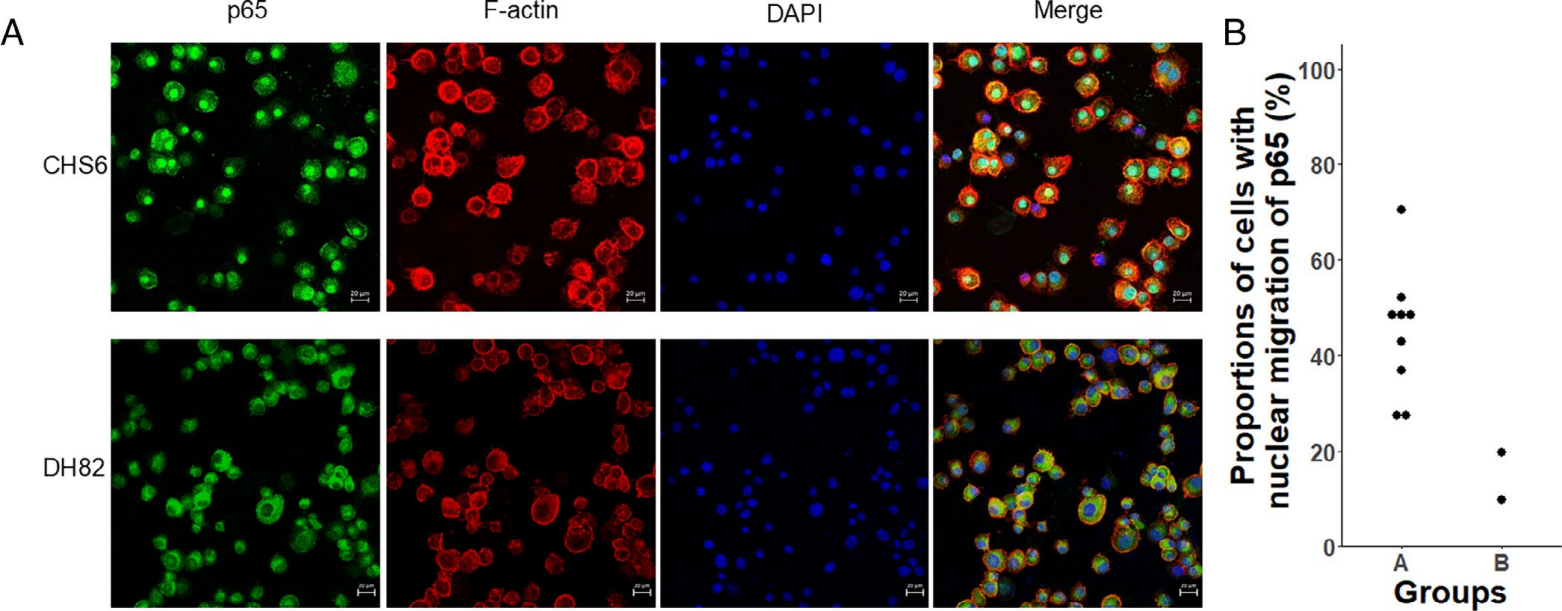
(A) Results of fluorescent immunocytochemistry in the two representative cell lines included in Group A or B. Top: CHS6, A cell line classified as Group A, bottom: DH82, A cell line classified as Group B, green: P65, Red: F‐Actin, blue: DAPI, scale bar: 20 μm. (B) Comparison of proportions of cells with nuclear translocation of p65 protein between the groups.

The obtained results indicated the potential associations of the activation status of the ERK, Akt, and NF‐κB pathways with the differences in GEPs between Group A and B. Figure 3 shows the data obtained by a reinterpretation of the results of our previous study that investigated the amounts of phosphorylated ERK and phosphorylated Akt proteins in the CHS cell lines [12]. The result showed that no apparent difference was observed in the amounts of these proteins between Group A and B. Additionally, no apparent difference was observed in the sensitivities to dasatinib, trametinib, and ponatinib, an agent targeting FGFR1 and previously reported to have antitumor effects in CHS cell lines, between Groups A and B when reinterpreting the results of the same previous study [17]. On the other hand, we found that the activation status of the NF‐κB pathway was different between the two groups. Figure 4A shows the representative results of immunocytochemistry for p65 protein, one of the important constituents of the NF‐κB pathway, in CHS6 and DH82, which were included in Group A and Group B, respectively. In CHS6, large amounts of p65 were translocated into the nucleus, whereas not in DH82. Figure 4B shows the proportion of cells with nuclear translocation of p65 protein in each cell line, and those in cell lines of Group A were higher than those in the cell lines of Group B.

### 3.5 Comparisons of the CCL5 Expressions Between Group A and B

**FIGURE 5:**
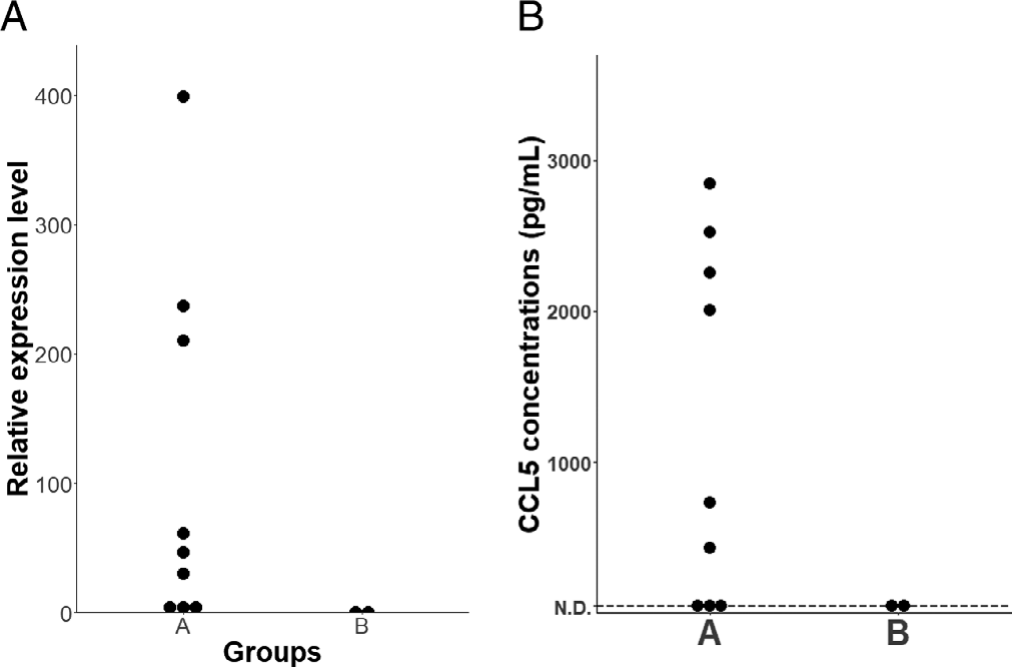
(A) Expression level of the *CCL5* gene in each canine histiocytic sarcoma cell line classified as Group A or B compared with the expression level in DH82. (B) Comparison of the amounts of CCL5 protein in the cell culture supernatant of canine histiocytic sarcoma cell lines between Group A and B.

NF‐κB pathway regulates the expressions of various chemokines including CCL5 [41, 42, 43]. Therefore, the expression level of CCL5 was compared between Groups A and B to validate the difference in the activation status of NF‐κB pathway functionally. As a result, mRNA expression levels of *CCL5* were shown to be increased in cell lines of Group A compared to those of Group B (Figure 5A). Then, we proceeded to quantify the amounts of CCL5 protein in the cell culture supernatant of each cell line via ELISA based on the availability of a specific antibody against canine CCL5. As shown in Figure 5B, six of the nine cell lines of Group A showed concentrations of 400 pg/mL or higher of CCL5, while both cell lines of Group B showed their concentrations below the lower limit of detection.

### 3.6 Association of Anti‐Proliferative Effects of NF‐κB Specific Inhibitor With Nuclear Translocation of p65 Protein

**FIGURE 6:**
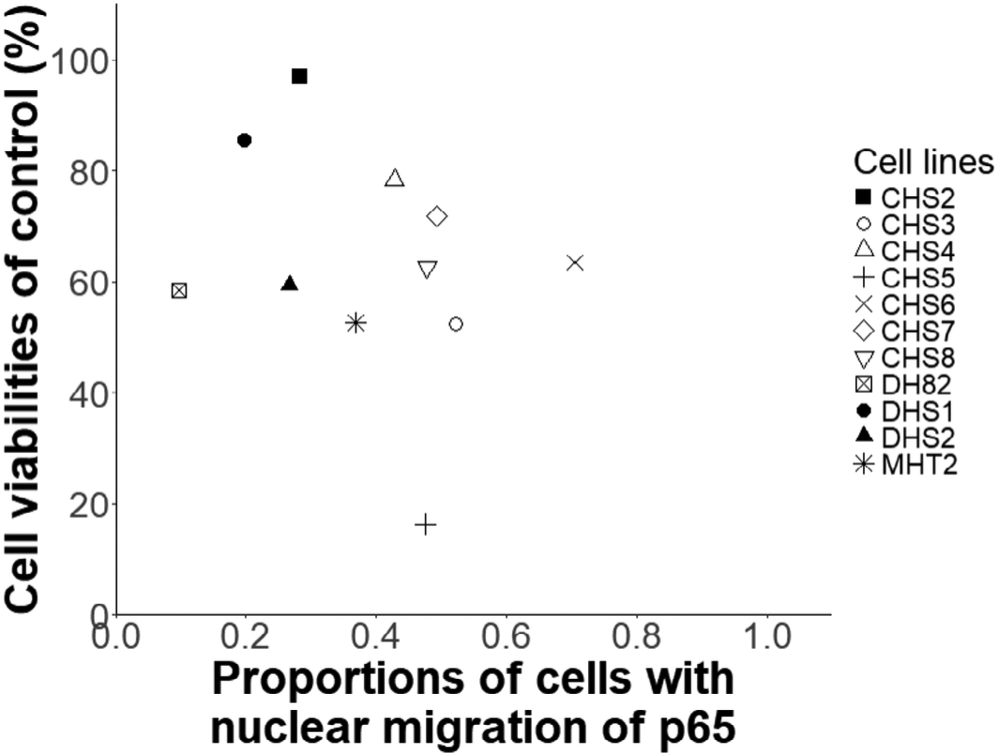
Association of proportions of cells with nuclear translocation of p65 protein with the cell viability after treatment with 1 μM of QNZ, an NF‐κB specific inhibitor, compared with 0.1% DMSO control.

Based on the observations above, we examined the anti‐proliferative effects obtained by the inhibition of the NF‐κB pathway in CHS cell lines. When the cells were treated with an NF‐κB pathway‐specific inhibitor, QNZ, at a concentration of 1 μM, CHS cell lines except for CHS5 exhibited cell viability of more than 50% compared to respective control cells. Then, we examined the association of the anti‐proliferative effect of QNZ with the proportion of cells with nuclear translocation of the p65 protein in each cell line (Figure 6). Though not statistically significant, CHS cell lines with higher proportions of cells showing nuclear translocation of p65 tended to have lower cell viability after treatment with QNZ (correlation coefficient: −0.300), indicating the possible involvement of the NF‐κB pathway in the regulation of cell proliferation in CHS cells.

## 4 Discussion

In this study, we aimed to classify CHS cells based on molecular abnormalities, and we revealed that the significant differences in GEPs divided the CHS cell lines into the two groups. Furthermore, we found that the activation status of the NF‐κB pathway was the hallmark to differentiate the groups.

In the field of human medicine, various tumours are subclassified based on their underlying molecular pathology, enabling the implementation of treatments that are tailored to the specific molecular characteristics of each tumour cell type. For example, in breast cancer, patients with high expression levels of oestrogen receptors are treated with hormone therapy. Similarly, in lymphoma, the comparison of GEPs led to the classification of patients into germinal center B‐cell‐like and activated B‐cell‐like diffuse large B‐cell types with different clinical characteristics [44, 45]. In dogs, subclassifications based on molecular abnormalities have not been established in any of the tumour types in detail, and the treatments are standardised based on the clinical diagnosis. Also, in CHS, cases are classified based on anatomical distributions and cell of origin: disseminated, localised, and hemophagocytic types [1], and attempts to classify CHS cases according to molecular abnormalities have been still limited [18, 19, 20]. This study was conducted to elucidate the heterogeneities in molecular pathologies of CHS in terms of GEPs and to obtain the basic knowledge for the establishment of the precision medicine for CHS.

The results of this study suggested that CHS cell lines could be classified into the two groups based on GEPs, and the activation status of the NF‐κB pathway was different between the two groups considering the differences in the proportions of cells with p65 nuclear translocations. The NF‐κB pathway is an intracellular signalling pathway that regulates both innate and adaptive immune responses [46, 47]. In tumour cells, this pathway has been reported to be activated, promoting cell survival and inhibition of apoptosis [46, 47]. Actually, it has been recently reported that approximately half of the CHS cases exhibited the activation of the NF‐κB pathway, indicating the heterogeneity of CHS cases in terms of its activation [40]. Furthermore, a proportion of CHS cases could harbour predisposing variants in the predisposing locus of chromosome 19 leading to an increase in TNFAIP6 expression resulting in the suppression of the NF‐κB pathway [48, 49]. Considering these findings, it is possible that the molecular pathology of CHS cases can also be divided into two groups based on the activation status of the NF‐κB pathway. However, since the present study included only cell lines derived from CHS cases, it is necessary to investigate whether the classifications based on molecular abnormalities, specifically the activation status of the NF‐κB pathway, can be applied to clinical CHS cases.

NF‐κB pathway is closely related to the regulations of immune responses such as chemokine production. In the present study, the mRNA expressions of chemokines *CCL3*, *CCL4*, *CCL5*, and *CCL7* and CCL5 protein expressions were increased in CHS cell lines included in one of the subtypes, Group A. This observation confirmed the functional differences in the activated status of the NF‐κB pathway between the groups. CCL5 is a member of the chemokine family produced by immune cells, which controls cell migration [50]. It is known that CCL5 promotes tumour metastasis by inhibiting the ubiquitin degradation of HIF1α in human liver cancer [51], and that CCL5 expression is increased in human oesophageal cancer [52]. It is also known that CCL5 expression induces tumour‐associated fibroblasts in the tumour microenvironment, thereby promoting tumour cell proliferation, and that high CCL5 expression is generally associated with poor prognosis [52]. It was also shown that the expressions of CCL2 were increased in CHS that occurred in Bernese Mountain dogs compared to those in clinical healthy ones [53], although no read was mapped to the *CCL2* gene locus in the reference genome used for RNA‐sequencing in the present study because this gene was not annotated in Ensembl 104, which was used in this study. Therefore, further studies are needed to elucidate the roles of these chemokines in the molecular pathology of CHS tissues and their associations with clinical features of CHS cases.

Contrary to our expectations, this study revealed that the inhibition of the NF‐κB pathway did not completely suppress the proliferations of CHS cells, although cell lines with higher proportions of cells showing nuclear translocation of p65 tended to have lower cell viability after treatment with the NF‐κB inhibitor. It was possible that the inhibitor might not be fully effective to suppress this pathway in CHS cells or alternative intracellular signalling pathways might play more important roles to regulate the cell proliferations. We previously revealed that Akt and ERK pathways were commonly activated in CHS cells and these pathways were shown to be involved in the regulations of cell proliferation [17]. Thus, it is unclear if the treatments targeting this pathway alone would be effective in one of the CHS subtypes, and we need to investigate the appropriate treatments for each CHS subtype.

In this study, we focused on the differences in the activation status of the NF‐κB pathway between the subgroups based on the results of enrichment analysis using RNA sequencing data. However, the results also indicated the differences in gene expressions associated with several other pathways between the groups. For example, KEGG pathway analysis extracted “phagosomes” as the pathway enriched with DEGs with the lowest FDR. CHS originates from dendritic cells or macrophages [1], which are known to possess phagosomes [54, 55]. Notably, macrophages retain strong phagocytic activity even after maturation, while dendritic cells lose this activity upon maturation [56]. Although one of the cell lines used in the present study, DH82, has been reported to be derived from macrophages [25, 57], the cell origins of the other cell lines remain unclear. Therefore, it is possible that the molecular differences observed among CHS cell lines could reflect the differences in the maturation stages of their cells of origin. For example, CCL3, SPP1, CD163, MSR1, and CLEC7A, extracted as DEGs upregulated in Group A, are known to be markers of alveolar macrophages and monocyte‐derived macrophages [58]. On the other hand, CD200 and AXL, upregulated in Group B, are reported to be dendritic markers [59]. Detailed investigations of the expression profiles of these differentiation markers may reveal the cell origin of the respective cell line and help to understand more detailed molecular pathogenesis.

In conclusion, twelve CHS cell lines were classified into two groups based on GEPs in this study, and the hallmark of the classification appeared to be differences in the activation status of the NF‐κB pathway. However, it is unclear if the treatments targeting this pathway alone would be effective in one of the CHS subtypes, and we need to investigate the appropriate drugs for each subtype to establish precision medicine for CHS cases.

## Conflicts of Interest

The authors declare no conflicts of interest.

## Supporting information


**Fig. S1.** Volcano plot with differentially expressed genes (DEGs) extracted by the comparisons of gene expression profiles between Groups A and B. The points coloured in blue represent the DEGs (283 genes) whose expressions were increased in Group A compared to Group B. The points coloured in red represent the DEGs (392 genes) whose expressions were decreased in Group A compared to Group B.

**Table S1.** RNA integrity numbers, total read counts, and mapping rates obtained by RNA‐sequencing analysis.
**Table S2.** Primer pairs used for reverse transcirpt quantitative polymerase chain reaction.
**Table S3.** Differentially expressed genes upregulated in Group A compared to Group B.
**Table S4.** Differentially expressed genes downregulated in Group A compared to Group B.
**Table S5.** Biological process terms that were enriched with differentially expressed genes in gene ontology analysis.
**Table S6.** Cellular component terms that were enriched with differentially expressed genes in gene ontology analysis.
**Table S7.** Molecular function terms that were enriched with differentially expressed genes in gene ontology analysis.
**Table S8.** Pathways enriched with differentially expressed genes in kyoto encyclopedia of genes and genomes (KEGG) pathway analysis.

## Data Availability

The datasets used and analyzed in RNA sequencing analysis in the present study are available at the DDBJ Sequenced Read Archive repository with accession number PRJDB17594. The data that support the findings of this study are available from the corresponding author upon reasonable request.
