# Supplementary figures and images for: Molecular Classification Based on the Gene Expression Profiles in Canine Histiocytic Sarcoma Cells
Source: Vet Comp Oncol. 2025 Jun 11;23(3):465–75. doi: 10.1111/vco.13071 (PMC12378086; doi:10.1111/vco.13071)

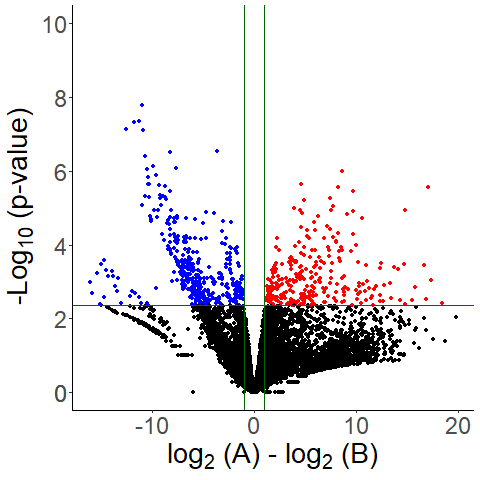

Supplement: Supplementary file 1 — Fig. S1. Volcano plot with differentially expressed genes (DEGs) extracted by the comparisons of gene expression profiles between Groups A and B. The points coloured in blue represent the DEGs (283 genes) whose expressions were increased in Group A compared to Group B. The points coloured in red represent the DEGs (392 genes) whose expressions were decreased in Group A compared to Group B. [file VCO-23-465-s002.tiff]
